# Pregnancy incidence and associated factors among HIV-infected female adolescents in HIV care in urban Côte d'Ivoire, 2009–2013

**DOI:** 10.3402/gha.v9.31622

**Published:** 2016-08-12

**Authors:** Shino Arikawa, Tanoh Eboua, Kouadio Kouakou, Marie-Sylvie N'Gbeche, Madeleine Amorissani-Folquet, Corinne Moh, Ursula Belinda Amoussou-Bouah, Patrick Ahuatchi Coffie, Renaud Becquet, Valériane Leroy, for the Pediatric IeDEA West Africa Working Group

**Affiliations:** 1 Inserm, Centre de recherche Inserm U1219, Bordeaux, France; 2 Institut de Santé Publique Epidémiologie Développement (ISPED), Université Bordeaux, Bordeaux, France; 3 Centre Hospitalier Universitaire Yopougon, Abidjan, Côte d'Ivoire; 4 Centre Intégré de Recherches Biocliniques, Abidjan, Côte d'Ivoire; 5 Centre de Prise en charge de Recherche et de Formation, Abidjan, Côte d'Ivoire; 6 Centre Hospitalier Universitaire Cocody, Abidjan, Côte d'Ivoire; 7 Centre de Prise en charge de Recherche et de Formation, Abidjan, Côte d'Ivoire; 8 Centre Hospitalier Universitaire Yopougon, Abidjan, Côte d'Ivoire; 9 Programme PACCI, Abidjan, Côte d'Ivoire; 10 Service des Maladies Infectieuses et Tropicales, CHU de Treichville, Abidjan, Côte d'Ivoire; 11 Inserm, Centre de recherche Inserm U1027, Université Paul Sabatier Toulouse 3, Toulouse, France

**Keywords:** HIV, adolescent, pregnancy, epidemiology, risk factors, Africa

## Abstract

**Objective:**

Adolescents living with HIV are sexually active and engaged in risky sexual behaviors. Knowledge on how and to what extent adolescents in HIV care are affected by pregnancy is needed so as to adopt better preventive services. We estimated 4-year pregnancy incidence and correlates among HIV-infected female adolescents in HIV care in urban Côte d'Ivoire.

**Design:**

We conducted retrospective analysis of a pediatric prospective cohort of the International epidemiological Databases to Evaluate AIDS (IeDEA) West Africa Collaboration. Female patients with confirmed HIV infection aged 10–19 years, having at least one clinical visit in 2009 to health facilities participating in the pediatric IeDEA West African cohort in Abidjan, Côte d'Ivoire, were included. Data on incident pregnancies were obtained through medical records and interviews with health professionals. Pregnancy incidence rate was estimated per 100 person-years (PY). Poisson regression models were used to identify factors associated with the first pregnancy and provided incidence rate ratios (IRR) with 95% confidence intervals (CI).

**Results:**

In 2009, 266 female adolescents were included, with a median age of 12.8 years (interquartile range, IQR: 10.0–15.0), CD4 cell counts of 506 cells/mm^3^ (IQR: 302–737), and 80% on antiretroviral treatment. At the 48th month, 17 new pregnancies were reported after 938 PY of follow-up: 13 girls had one pregnancy while 2 had two pregnancies. Overall incidence rate of pregnancy was 1.8/100 PY (95% CI: 1.1–2.9). High incidence was observed among those aged 15–19 years: 3.6/100 PY (95% CI: 2.2–5.9). Role of maternal death in the risk of pregnancy was at the limit of statistical significance (adjusted IRR: 3.1, 95% CI: 0.9–11.0; ref. non-maternal orphans).

**Conclusions:**

Incidence of pregnancy among HIV-infected adolescents in care aged 15–19 years reached a level observed in adult cohorts in Sub-Saharan Africa. Health personnel in pediatric care have to intensify their efforts to provide more realistic and age-adapted reproductive health services to meet the needs of adolescent patients already confronting issues of sexuality. Vulnerability of maternal orphans merits further investigation.

## Introduction

According to the World Health Organization (WHO), approximately one in six persons in the world is an adolescent (1). The absolute number of adolescents is rising on a global scale, reaching an unprecedented level of 1.2 billion in 2010 (2). Among many health challenges that adolescents face, issues related to pregnancy and childbirth should be given high priority particularly within the context of lower income countries. Sub-Saharan African countries show the highest birth rate among female adolescents (number of births per 1,000 women aged 15 and 19 years), 123 births per 1,000, as compared with 49 per 1,000 girls worldwide (2, 3). The rate in Sub-Saharan Africa has not dropped since 1990 while other regions have seen a marked decline (2, 3). The West African region is particularly hit hard by this phenomenon. In Niger, Mali, and Côte d'Ivoire, respectively, 51, 46, and 29% of women aged between 20 and 24 years reported having given birth before the age of 18 years (2). Pregnancies in girls aged below t15 years were not negligible either as they were reported by 13 and 6% of women in Sierra Leone and in Côte d'Ivoire, respectively (4).

In 2013, 2.1 million adolescents were living with HIV worldwide, with 90% of them in Africa (5). Studies have reported that sexual behaviors of HIV-infected adolescents were not significantly different from those of non-infected counterparts (3, 6, 7). Adolescents living with HIV are often sexually active and engaged in risky sexual behaviors including unprotected sex or having multiple partners (7). Poor negotiation skills, fear of rejection, and low self-esteem make them particularly vulnerable to unprotected sex. HIV status disclosure to sexual partners is extremely rare (8). It is not surprising therefore that pregnancy in this population is not a rare phenomenon. A cross-sectional study in Kenya revealed that 50% of female HIV-infected adolescents have had their first pregnancy before the age of 17 (9). A study in Uganda also showed that the incidence of pregnancy of perinatally infected adolescents was not significantly different from those not infected (10).

To date, pregnancy incidence has only been measured in adult patients. As a large number of adolescents living with HIV are becoming sexually active, knowledge on how and to what extent adolescents living with HIV are affected by pregnancy is needed. The objective of our study is therefore to estimate the incidence of pregnancy and its associated factors among HIV-infected female adolescents in HIV care in urban Côte d'Ivoire between 2009 and 2013. The results of our study will contribute to a better understanding of the burden of pregnancies and adoption of more targeted strategies to prevent unplanned pregnancies in this population.

## Methods

The International epidemiological Databases to Evaluate AIDS (IeDEA) initiative (www.iedea-hiv.org), launched in 2006, is a consortium of leading clinicians and epidemiologists. The present analysis was conducted in four health facilities participating in the pediatric IeDEA West African HIV cohort (pWADA) in Abidjan, Côte d'Ivoire, namely CIRBA, CePReF, Yopougon, and Cocody University Hospitals. HIV-infected children aged <10 years at the time of their HIV diagnosis were seen at least every 3 months according to national guidelines. All female patients with a confirmed HIV infection, who had at least one contact during the calendar year 2009 with one of the four pediatric pWADA clinics mentioned above, and aged 10–19 years at their first visit in 2009 were included in our study. Patients without any single follow-up visit during the study period were excluded from the analysis. Age, vital status of mother and father, weight, height, CD4 cell count, hemoglobin at the time of inclusion in the study, and the date of anti-retroviral therapy (ART) initiation (if on ART) were extracted from the pWADA database. When biological or clinical data were not recorded exactly on the date of the first visit in 2009 (i.e. baseline of our study), we used the most updated data within a range of 3 months.

Episodes of pregnancies were sought through multiple sources. In most cases, they were self-declared and recorded in patient's files. In addition to these written records, health care workers were asked to list all pregnancies which could have occurred between 2009 and 2013 and to provide detailed information about each case. The information provided by a given personnel was cross-checked by other staffs to ensure its validity. When the information on gestational age, date of delivery, pregnancy outcome, or a child's HIV status could not be found in medical records, health care workers phoned the patients or relevant services (adult or obstetric) to acquire necessary information. Pregnancy intention for the first pregnancy was assessed by health personnel over the phone with the London Measure of Unplanned Pregnancy (LMUP) (www.lmup.com/) for those still in follow-up in 2016. The LMUP is a psychometrically validated measure of pregnancy and planning/intention (11). In the present study, we used a French version of this tool which we had adapted to the local context with midwives in the study sites through a translation back translation method (12).

Each adolescent contributed to the denominator from the time they entered into the study until either 31 December 2013, date of the 20th birthday, date of death or loss-to-follow-up, date of transfer out, or date of first pregnancy, whichever occurred first. Loss to follow-up was confirmed when the patient did not report for any follow-up for at least 6 months, and for whom vital status could not be confirmed. Pregnant adolescents were right-censored at the estimated conception date and subsequently uncensored and re-included in analysis after a 15-month period in the case of a term live-birth. The 15 months of censoring was chosen based on 9 months of pregnancy and 6 months of post-partum abstinence, which is a common practice among women in Côte d'Ivoire (13). When a pregnancy was terminated by a spontaneous miscarriage or induced abortion, the adolescent was right-censored on the estimated date of conception and uncensored at the date of pregnancy interruption. Re-inclusion of adolescents with pregnancy episode allowed analysis of recurrent pregnancies. Delayed entry was allowed for those who were pregnant at baseline (*n*=2).

Baseline characteristics were described by median values with interquartile range (IQR) for continuous variables and frequencies for categorical variables, stratified according to the occurrence of pregnancy over the study period from 2009 to 2013. Pregnancy incidence was calculated per 100 person-years (PY) of follow-up with their 95% confidence intervals (95% CI). Poisson regression method was used to estimate the incidence rate of pregnancy according to baseline patient characteristics. Factors associated with the occurrence of first pregnancy were analyzed using Poisson regression. Immunodeficiency was defined as <350 cells/mm^3^ as per the 2010 WHO recommendations for ART initiation (14). Age group was computed as a time-dependent variable, summarizing for each girl the number of months contributing to the period of a given age category. Univariate analyses were run with all the covariables of interest. The variables included in multivariate analyses were selected at the threshold of *p*=0.25 through a stepwise descending method. In the final model, statistical significance was considered two-sided when *p*<0.05. Unadjusted incidence rate ratios (IRRs) and adjusted IRR (aIRR) are reported with their 95% CI. Data were entered into Microsoft Access 2003, and all the statistical analyses were performed using SAS version 9.3 (SAS Institute, Cary, NC).

## Results

**Fig. 1:**
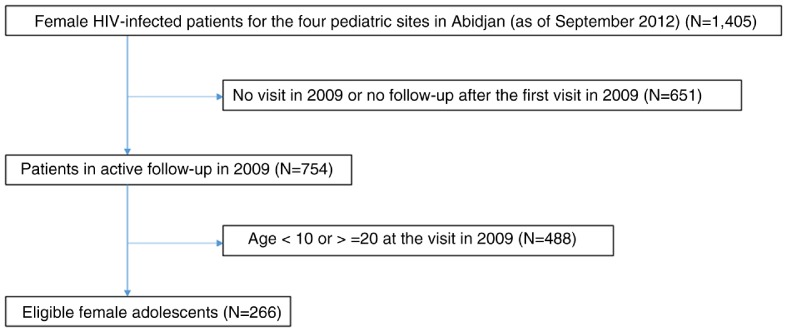
Flow chart of HIV-infected female adolescents eligible for the analysis.

In 2009, 266 female adolescents were included in the study. The flow chart of the patients’ selection is shown in Fig. 1. Their median age was 12.8 years (IQR: 10.0–15.0) at inclusion, and was higher for girls ever becoming pregnant during follow-up (14.0 years) than others (12.6 years) (Table 1).

**Table 1:** Baseline characteristics of HIV-infected female adolescents according to pregnancy status over a period of 48 months in Abidjan, Côte d'Ivoire (*n*=266)

	Ever becoming pregnant (*N*=15)		Never becoming pregnant (*N*=251)		Total (*N*=266)	
			
Variable	*n*	IQR	*n*	IQR	*n*	IQR
Age (years), median	14.0	13.0–16.2	12.6	11.3–14.9	12.8	10.0–15.0
CD4 (cells/mm^3^), median	432	246–739	507	302–737	506	302–737
	*n*	%	*n*	%	*n*	%
Center						
CEPREF	5	33.4	65	25.9	70	26.3
Cocody University Hospital	2	13.3	17	6.7	19	7.1
Yopougon University Hospital	6	40.0	129	51.4	135	50.8
CIRBA	2	13.3	40	16.0	42	15.8
Age category (years)						
<15	8	53.3	191	76.1	199	74.8
≥15	7	46.7	60	23.9	67	25.2
Maternal orphan at baseline						
Yes	12	80.0	127	50.6	139	52.3
No	3	20.0	82	32.7	85	32.0
Unknown	0	0.0	42	16.7	42	15.7
Paternal orphan at baseline						
Yes	4	26.7	94	37.4	98	36.8
No	10	66.7	111	44.2	121	45.5
Unknown	1	6.6	46	18.4	47	17.7
Weight-for-age (z-score)						
Normal	13	86.8	170	67.7	183	68.8
Moderate undernutrition (<−2 SD)	1	6.6	27	10.8	28	10.5
Severe undernutrition (<−3 SD)	1	6.6	54	21.5	55	20.7
Height-for-age (z-score)						
Normal	11	73.3	157	62.6	168	63.2
Moderate stunting (<−2 SD)	3	20.0	34	13.6	37	13.9
Severe stunting (<−3 SD)	0	0.0	44	17.5	44	16.5
Unknown	1	6.7	16	6.3	17	6.4
Anemia (defined as <12 g/dl)						
Yes	10	66.7	175	69.7	185	69.6
No	5	33.3	73	29.1	78	29.3
Unknown	0	0	3	1.2	3	1.1
CD4 (cells/mm^3^)						
<200	3	20.0	44	17.5	47	17.6
200–349	2	13.3	31	12.3	33	12.4
350–499	3	20.0	45	18.0	48	18.1
>500	7	46.7	131	52.2	138	51.9
BMI	4	26.7	158	63.0	162	61.0
Underweight (<18.5)						
Normal (18.5–25.0)	9	60.0	71	28.3	80	30.1
Overweight (≥25.0)	2	13.3	4	1.6	6	2.2
Unknown	0	0.0	18	7.1	18	6.7
On ART at baseline						
Yes	11	73.3	200	79.7	211	79.3
No	4	26.7	51	20.3	55	20.7

Girls younger than 15 years (age at baseline) accounted for almost 75% of the study participants. Orphanhood was frequently observed in this population regardless of pregnancy status. In particular, maternal death was a common event, affecting one in two girls overall. Adolescents ever becoming pregnant had lost their mothers more frequently than those experiencing no pregnancy (80.0% vs. 32.7%). At baseline, most of them were anemic (69.6%) and 31.2% were underweight (defined as weight-for-age z-score <−2 SD). As much as 40% of the study sample presented a certain degree of growth failure (moderate and severe stunting was defined as height-for-age z-score <−2 and <−3 SD, respectively). Baseline median CD4 cell counts were 506 cells/mm^3^ (IQR: 302–737). Around 80% of the adolescents were on ART at inclusion in the study.

Over the study period, 17 incident pregnancies (first and recurrent) were reported: 13 adolescents experienced one pregnancy and 2 experienced two pregnancies. No pregnancy occurred among adolescents younger than 13 years (age at time of pregnancy). The median age at first pregnancy was 17.7 years (IQR: 16.5–18.7). Table 2 shows the number of pregnancies, person-years observed, and incidence of pregnancy per 100 persons-years according to the age at pregnancy occurrence (time-dependent variable). The incidence increases considerably with age, particularly from age 17. Probably due to the small sample size in our study, no pregnancy events were observed among girls aged 16 years.

**Table 2:** Number of pregnancies, person-years observed, and incidence of pregnancy per 100 PY according to the age at pregnancy occurrence (time-dependent variable) among HIV-infected female adolescents in Abidjan, Côte d'Ivoire (*n*=266, 938 PY)

Age (time dependent)	No. of pregnancies	PY	Pregnancy incidence/100 PY (95% CI)
10	0	21.1	0.0
11	0	69.5	0.0
12	0	110.6	0.0
13	0	138.8	0.0
14	1	153.6	0.7 (−0.6–1.9)
15	4	147.7	2.7 (0.1–5.3)
16	0	112.0	0.0
17	6	84.1	7.1 (1.6–12.6)
18	3	62.6	4.8 (−0.5–10.1)
19	3	38.1	7.9 (0.7–16.4)

PY= person-years.

Only one out of 15 adolescents was in a formal marital relationship at the time of first pregnancy while others were single (neither in formal marriage nor cohabiting unions). Excluding the three adolescents who were still pregnant at endpoint, the majority (*n*=9) of the 14 pregnancies resulted in live births (64.3%). One spontaneous miscarriage (7.1%) and two induced abortions (21.4%) were observed. The outcomes of the remaining two pregnancies could not be ascertained. All of the nine HIV-exposed children with birth records received a confirmation of non-infection by HIV at the end of the mother-to-child exposition period reflecting the fact that all of these adolescent mothers were on ART. Pregnancy intention was assessed among 6 out of the 15 adolescents who became pregnant. Other nine had either died (*n*=2) or were lost-to-contact (*n*=7) at the time of this survey. None of these adolescents declared having had any intention of getting pregnant. Two adolescents described the arrival of their pregnancies ‘at the wrong time’ whereas four others said ‘not quite the right time’. Four adolescents had never discussed having children with their partner while two others said that they have had discussions but never agreed that she would be pregnant.

Incidence rate of pregnancy was estimated at 1.6 per 100 PY for the first pregnancy (95% CI: 0.7–3.5) and 1.8 per 100 PY (95% CI: 1.1–2.9) overall. Table 3 details the incidence rate of pregnancy according to baseline and time-adjusted characteristics of the 266 adolescents included in the study. The incidence rate in older adolescents (aged 15–19 years) reached 3.6 per 100 PY (95% CI: 2.2–5.9) when multiple pregnancies were taken into account. The pregnancy rate of maternal orphans at baseline was 2.7 per 100 PY (95% CI: 1.5–4.6), being slightly higher than non-orphans, although the difference was not statistically significant. As presented in Table 4, adolescents aged between 15 and 19 years were 14.2 times more likely to experience their first pregnancy than those aged 10–14 years (95% CI: 1.9–108.2). Maternal orphans seemed to be at higher risk compared with non-orphans; however, the difference was at the limit of statistical significance (aIRR: 3.1, 95% CI: 0.9–11.0). It is important to note however that we grouped together the observation time of adolescents whose mothers were known to be alive and those having no information on mother's vital status in order to maintain the maximum number of study samples. When a sensitivity analysis was performed assuming that those with no information were indeed maternal orphans, the difference no longer reached statistical significance (IRR: 2.3, 95% CI: 0.6–8.1). There was no association between baseline levels of nutritional status, hemoglobin, CD4 cell counts, ART use, and father's vital status with occurrence of the first pregnancy.

**Table 3:** Incidence rates of pregnancy among HIV-infected female adolescents in Abidjan, Côte d'Ivore (*n*=266)

Characteristic	No. of first pregnancies/PY	Incidence of first pregnancy/100 PY (95% CI)	Overall no. of pregnancies/PY	Pregnancy incidence/100 PY (95% CI)
Global	15/938.0	1.6 (0.7–3.5)	17/939.0	1.8 (1.1–2.9)
Center				
CEPREF	5/260.7	1.9 (0.8–4.6)	5/260.7	1.9 (0.8–4.6)
Cocody University Hospital	2/68.0	2.9 (0.7–11.8)	2/68.0	2.9 (0.7–11.8)
Yopougon University Hospital	6/452.4	1.3 (0.6–3.0)	8/453.4	1.8 (0.9–3.5)
CIRBA	2/156.9	1.3 (0.3–5.1)	2/156.9	1.3 (0.3–5.1)
Age (time adjusted) (years)				
<15	1/493.5	0.2 (0.0–1.4)	1/493.5	0.2 (0.0–1.4)
≥15	14/444.5	3.2 (1.9–5.3)	16/445.5	3.6 (2.2–5.9)
Maternal orphan at baseline				
Yes	12/490.1	2.5 (1.4–4.3)	13/491.1	2.7 (1.5–4.6)
No	3/340.8	0.9 (0.3–2.7)	4/340.8	1.8 (0.1–3.1)
Unknown	0/107.1	*	0/107.1	*
Paternal orphan at baseline				
Yes	4/352.1	1.3 (0.5–3.4)	6/352.1	1.1 (0.4–3.0)
No	10/460.8	2.2 (1.2–4.0)	10/461.8	2.2 (1.2–4.0)
Unknown	1/125.1	2.4 (0.3–17.0)	1/125.1	2.4 (0.3–17.0)
Weight-for-age (z-score)				
Normal	13/673.9	1.9 (1.1–3.3)	15/674.9	2.2 (1.3–3.7)
Moderate undernutrition (<−2 SD)	1/102.8	1.0 (0.1–6.9)	1/102.8	1.0 (0.1–6.9)
Severe undernutrition (<−3 SD)	1/161.3	0.6 (0.1–4.4)	1/161.3	0.6 (0.1–4.4)
Height-for-age (z-score)				
Normal	11/609.0	1.81 (1.0–3.3)	13/610.0	2.1 (1.2–3.7)
Moderate stunting (<−2 SD)	3/142.2	2.1 (0.7–6.5)	3/142.2	2.1 (0.9–6.5)
Severe stunting (<−3 SD)	0/126.4	*	0/126.4	*
Unknown	1/60.4	1.7 (0.2–11.8)	1/60.4	1.7 (0.2–11.8)
Anemia (defined as <12 g/dl) at baseline				
Yes	10/653.6	1.5 (0.6–3.9)	10/653.6	1.5 (0.6–3.9)
No	5/280.3	1.8 (0.5–6.6)	7/281.3	2.5 (0.5–13.2)
Unknown	0/4.1	*	0/4.1	*
CD4 (cells/mm^3^) at baseline				
<350	5/241.1	2.1 (0.9–5.0)	6/242.1	2.5 (1.1–5.5)
≥350	10/696.9	1.4 (0.8–2.7)	11/696.9	1.6 (0.9–2.9)
On ART at baseline				
Yes	11/755.8	1.5 (0.8–2.6)	12/755.8	1.6 (0.9–2.8)
No	4/182.2	2.2 (0.8–5.9)	5/183.2	2.7 (1.1–6.6)

PY= person-years.

* Not estimated due to the absence of events in this category.

**Table 4:** Factors associated with the first incidence of pregnancy among HIV-infected female adolescents in Abidjan, Côte d'Ivoire (*n*=266, 938 PY)

		Univariate models		Multivariate model	
			
Variable	No. of first pregnancies/PY	Crude IRR (95% CI)	*p*	Adjusted IRR (95% CI)	*p*
Center			0.78		
CEPREF	5/260.7	Ref.			
Cocody University Hospital	2/68.0	1.5 (0.3–7.9)			
Yopougon University Hospital	6/452.4	0.7 (0.2–2.3)			
CIRBA	2/156.9	0.7 (0.1–3.4)			
Age (time adjusted) (years)			0.01		<0.01
<15	1/493.5	Ref.		Ref.	
≥15	14/444.5	15.5 (2.0–118.2)		14.2 (1.9–108.2)	
Maternal orphan at baseline			0.03		0.05
Yes	12/490.1	3.7 (1.0–13.0)		3.1 (0.9–11.0)	
No/unknown	3/447.9	Ref.		Ref.	
Paternal orphan at baseline			0.37		
Yes	4/352.1	0.6 (0.2–1.9)			
No/unknown	11/585.9	Ref.			
Underweight (weight-for-age z-score <−2SD)					
Yes	2/264.0	Ref.	0.17		
No	13/674.0	2.6 (0.6–11.3)			
Stunting (height-for-age z-score <−2SD)					
Yes	4/329.1	Ref.	0.49		
No/unknown	11/608.9	1.5 (0.5–4.7)			
Anemia (hemoglobin <12 g/dl) at baseline					
Yes	10/653.6	0.9 (0.2–4.3)	0.87		
No/unknown	5/284.4	Ref.			
CD4 (cells/mm^3^) at baseline			0.50		
<350	5/241.0	Ref.			
≥350	10/697.0	0.7 (0.2–2.0)			
On ART at baseline			0.50		
Yes	11/755.9	0.7 (0.2–2.1)			
No unknown	4/182.1	Ref.			

ART=anti-retroviral therapy; IRR=incidence rate ratio; PY=person-years.

## Discussion

To our knowledge, this study reported for the first time the incidence of pregnancy and associated factors among HIV-infected adolescents within a pediatric cohort. Based on the data from different sources, the episodes of pregnancy during 2009 and 2013 were exhaustively sought, allowing a fairly accurate estimate of pregnancy rate among HIV-infected girls.

One of the most important findings in our study is that the pregnancy incidence rate among adolescents aged 15–19 years reached as high as 3.6 per 100 PY, which is comparable with that of adult women on ART. Westreich et al. and Myer et al. reported the rates of 5.2 (95% CI: 4.8–5.5) and 3.3 per 100 PY (95% CI: 2.6–4.2), respectively, in adult cohorts in South Africa (15, 16). In addition, the IeDEA adult cohort in West Africa reported the incidence rates of 4.8 (4.4–5.2) and 4.3 (4.0–4.6) among women aged 25–29 and 30–34 years, respectively (17). This clearly highlights the magnitude of pregnancy-related issues encountered by adolescents in HIV care. The results of our assessment using the LMUP have shown that six of the observed pregnancies were unintended. Due to death and loss of contact, we were unable to ascertain pregnancy intention in the majority of cases; however, we still believe that these pregnancies were likely to be unintended or at least unplanned, given the fact that these adolescents were not in a stable relationship at the time of pregnancy and that utilization of any modern family planning methods by adolescents is extremely low in general in Côte d'Ivoire (13). In fact, this view was echoed by health personnel through oral communication who were in close and regular contact with the adolescent patients.

One might think that HIV-infected adolescents in care program have access to appropriate sexual and reproductive health services and therefore are at lower risk of unwanted pregnancies and secondary transmission of HIV (18). Nonetheless, studies showed that access to family planning services of HIV-infected individuals is not satisfactory in general (8), and is sometimes hindered by healthcare staff's negative attitude (10, 19). Cultural disapproval of adolescent sexuality in general remains strong, which can be even more apparent when it comes to adolescents living with HIV (9). Our data clearly showed the unmet needs of sexual and reproductive health services in patients in late adolescence where high pregnancy incidence was observed. Given their long-term and regular contacts with adolescent patients, healthcare providers in pediatric service have an unique opportunity to meet their needs. Health personnel in pediatric care should be more active in providing age-adapted sexual and reproductive health services including access to contraceptives. Use of language heavily centering on abstinence or delaying sexual debut should be avoided. Adolescents with a desire or intention to become pregnant should not be judged or treated differently but should be supported to make an informed decision, as pregnancy in women living with HIV, regardless of age, should be well-planned so as to reduce the risk for her own health and of transmission of HIV to her sexual partner and to her own child. In such cases, adherence to ART, disclosure of HIV status to sexual partner, and maintenance of a healthy life style (nutrition, avoidance of tobacco, and alcohol consumption) should constitute one of the key messages to be delivered to adolescent patients.

Our multivariate analysis of correlates has suggested that maternal orphans at baseline might have a higher risk of pregnancy compared with non-orphans. Vulnerability of orphans to sexual risks has been investigated extensively in Sub-Saharan Africa, and there is a body of evidence on the high risk encountered by this population (20–22). Our results should be interpreted with caution however due to the presence of potential bias involved in classification of the study samples. As our sensitivity analysis has demonstrated, the risk of first pregnancy faced by maternal orphans could have been overestimated. Maternal death however is an event to which health personnel pay particular attention in our study setting, and it is unlikely that it remains unnoticed or unrecorded if it occurs. While improved data should be used to confirm this association, health care staff should take additional care of this group of adolescents, especially in relation to their psycho-social support. Clinical and immunological indicators including nutritional status, baseline level of hemoglobin, CD4 counts, or ART use were not associated with pregnancy incidence in our study as seen in adult HIV cohorts (15, 17, 23, 24).

Several limitations of our study must be mentioned. First, variables were selected from those existing in the pWADA database; thus, information relevant to pregnancy, such as contraception use, existence of partner, or marital status (for non-pregnant adolescents), was not available for analyses. Equally, due to inconsistent data collection and lack of standardized reporting format, exploration of socio-demographic and psycho-social variables relevant to the question was hindered. Second, underestimation of pregnancy rate might have occurred since most of incident pregnancy was based on patient's self-declaration. Some pregnancies might have thus remained undetected, especially in the case of induced abortions which are legally restricted in Côte d'Ivoire. Third, lack of information on the onset of menarche could have led to overestimation of the time at risk of the study population as a whole. Finally, the small sample size reduced statistical power.

## Conclusions

Our study provided a minimum estimate on the incidence of pregnancy and identified associated factors among female HIV-infected adolescents in care in Abidjan. Given various limitations of the study, the estimate made by this study could be regarded as rather conservative. The incidence rate in late adolescence has reached a level comparable with that of HIV-infected adult women in Sub-Saharan Africa. Bearing in mind evidence that many of these pregnancies are unintended, health professionals in pediatric care need to adapt more realistic and needs-based approach to address the issues of sexuality in adolescent patients. The study suggested a possible vulnerability of the maternal orphan although further analysis is needed to clarify its role.

## Paper context

Previous studies have reported that adolescents living with HIV are sexually active and engaged in risky sexual behaviors. However, pregnancy incidence has only been measured in adult patients to date. As the number of HIV-infected adolescents is increasing, knowledge on how and to what extent adolescents in HIV care are affected by pregnancy is needed.
